# On curiosity, neuroscience, and building interdisciplinary bridges: a conversation with Dr. Anna Roe

**DOI:** 10.1117/1.NPh.12.4.040401

**Published:** 2025-10-21

**Authors:** Christopher Moore

**Affiliations:** Brown University, Neuroscience Graduate Program, Providence, Rhode Island, United States

## Abstract

Dr. Anna Roe, Director of Translational Neuroscience at the Nathan Kline Institute for Psychiatric Research and Professor of Psychiatry and Neuroscience at New York University, discusses her journey as a neuroscientist, highlighting breakthroughs in brain plasticity, imaging, and the value of curiosity and mentorship.

**Figure:**
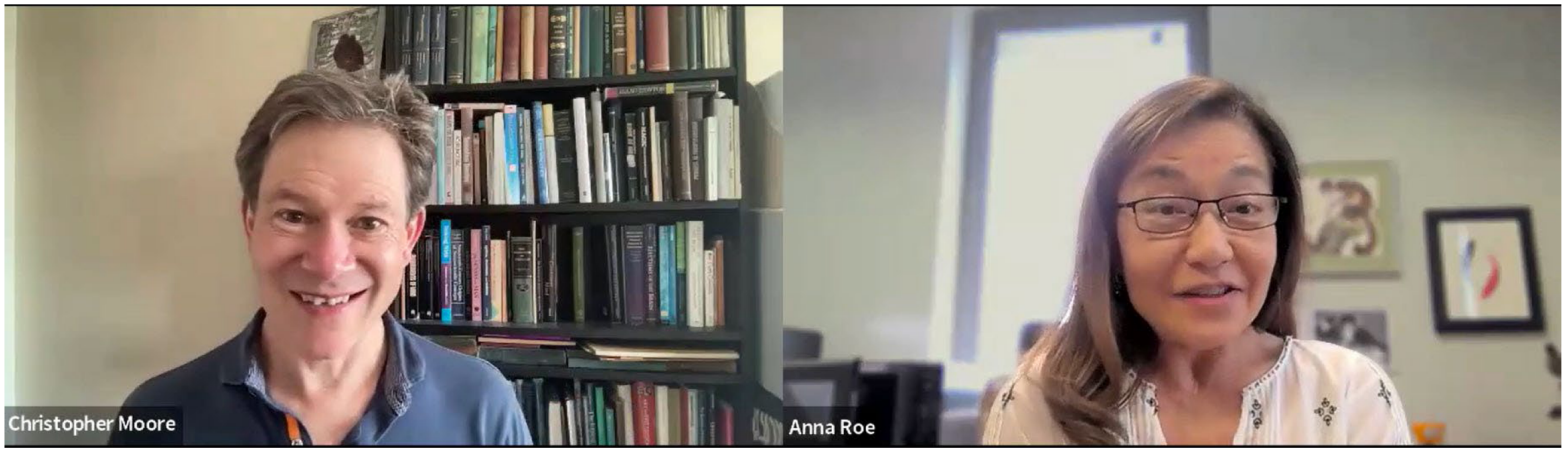
Christopher Moore (left) interviewed Anna Roe (right), Director of Translational Neuroscience at the Nathan Kline Institute for Psychiatric Research and Professor of Psychiatry and Neuroscience at New York University. View the interview video at https://doi.org/10.1117/1.NPh.12.4.040401.

In the world of neuroscience, few careers reflect the power of curiosity and interdisciplinary thinking as vividly as that of Dr. Anna Roe. From a childhood steeped in philosophical debate to pioneering work in cortical imaging and neuroplasticity, Roe’s journey is a testament to intellectual bravery and scientific imagination.

In a recent conversation with Dr. Roe, we explored her unconventional path into neuroscience, her groundbreaking discoveries, and the advice she offers to the next generation of researchers.

## 1 From Philosophy to Physiology

Roe didn’t begin her academic life in neuroscience—or even biology. “I never took neuroscience or psychology in school,” she recalls. “I thought I’d be a math person.” But a course in logic and philosophy at Harvard introduced her to Gödel, Turing, and Searle’s Chinese Room, igniting a fascination with the mind–brain relationship.

That spark led her to cold-call over 25 labs at Children’s Hospital in Boston. “I had no background in biology or biochemistry,” she says. “But Frost White gave me a chance.” That chance turned into a thesis and two published papers—her first steps into neuroscience.

## 2 Mentorship and the Power of Systems Thinking

Roe’s scientific trajectory was shaped by legendary mentors like David Hubel, Marge Livingstone, and Gordon Shepherd. “Good teachers can change your life,” she says. Coming from a math background, she was drawn to the brain as a system. “What kind of system is the brain?” she wondered. “People like Hubel and Wiesel inferred the hypercolumn structure of the cortex using just a single electrode. That blew my mind.”

Her fascination with cortical architecture led to a lifelong interest in modularity and plasticity—concepts that would define much of her research.

## 3 Rewiring the Brain: a Landmark Experiment

One of Roe’s most influential projects began at MIT, where she and Mriganka Sur explored whether the eye could drive cells in the auditory cortex. “It sounded like science fiction,” she laughs. But by rerouting retinal fibers into the auditory thalamus, they demonstrated that visual information could be processed in auditory regions—an astonishing display of cortical plasticity.

The work, published in *Science*, supported the idea of a “plug-and-play” cortical architecture. “It made me think that cortical columns are ubiquitous and adaptable,” Roe says.

## 4 Imaging the Awake Brain

Roe also pioneered the use of intrinsic signal optical imaging in awake, behaving animals—a notoriously difficult feat. “The signal is tiny—about 0.1%—and there’s a lot of noise,” she explains. Years of troubleshooting and computational refinement eventually paid off, opening new windows into real-time cortical function.

Her work revealed that even higher-order visual areas like V2 exhibit columnar organization and respond to percepts like illusory contours and depth—evidence for repeated, modular processing across the cortex.

## 5 Building Bridges in China

In 2012, Roe took a sabbatical to visit universities in China. A 20-minute presentation at Zhejiang University—delivered in Chinese—led to an unexpected offer: to found a new interdisciplinary neuroscience institute. “I was stunned,” she says. “Eventually, I had to choose between staying in the US or going to China full-time. I chose China.”

The institute grew into a full-fledged research center with ultrahigh field MRI, macaques, marmosets, and a vibrant team. “It was challenging, especially navigating a different administrative system, but incredibly rewarding.”

## 6 Mentorship, Compassion, and Curiosity

Looking back from her current role as Director of Translational Neuroscience at the Nathan Kline Institute for Psychiatric Research and Professor of Psychiatry and Neuroscience at New York University in New York, Roe’s advice to young researchers is both practical and profound:

Focus early: “Pick one area and do it really well.”

Apply for early career awards: “They help build momentum.”

Embrace feedback: “Reviewers are trying to help. Try to see your work through their eyes.”

Integrate life and science: “It’s not about choosing between family and career. It’s about integration.”

She also emphasizes the importance of mentorship and compassion. “I had a child during grad school, and my mentor was supportive. That taught me the value of flexibility in science.”

Roe’s curiosity remains a driving force: “I’m working on a pet project—developing a mathematical theory of the brain,” she shares. “We still don’t have a theoretical foundation for brain science. I hope we can one day reduce brain networks to fundamental principles and understand how intelligence arises.”

Roe’s story is a powerful reminder that science thrives on curiosity, resilience, and the courage to cross disciplinary boundaries. For early career neuroscientists, her journey offers both inspiration and a roadmap: follow your questions, seek great mentors, and never stop learning.

